# Synergistic interplay between UV and urban particulate matter exposure induces melanocyte senescence and contributes to human skin aging

**DOI:** 10.1038/s41598-025-28590-6

**Published:** 2025-12-29

**Authors:** Ines Martic, Lena Guerrero-Navarro, Elia Cappuccio, Amina Hassan, Brigitte Jenewein, Elsa Arcalis, Nina Hrapovic, Lene Visdal-Johnsen, Lieve Declercq, Pidder Jansen-Dürr, Maria Cavinato

**Affiliations:** 1 Institute for Biomedical Aging Research, Universität Innsbruck, Rennweg 10, 6020 Innsbruck, Austria; 2 Center for Molecular Biosciences Innsbruck (CMBI), Innsbruck, Austria; 3 Institut Für Pflanzenbiotechnologie Und Zellbiologie, University of Natural Resources and Life Sciences (BOKU), Vienna, Austria; 4 Scientific Research and Innovation, Oriflame Cosmetics AB, Stockholm, Sweden; 5 European Innovation Center, Proya Europe, Paris, France

**Keywords:** Cellular senescence, Environmental factors, Mitochondria, Skin aging, Pigmentation, Cell biology, Developmental biology, Diseases, Physiology

## Abstract

Extrinsic skin aging is driven by environmental factors, including ultraviolet (UV) radiation and air pollution. While melanocytes serve as key protectors against UV-induced damage, their role in aging, particularly through the process of senescence, remains underexplored. Here, we exposed human neonatal melanocytes and ex vivo skin explants to UV (UVA + UVB), urban particulate matter (UPM), and their combination (UV + UPM) to assess the effects on melanocyte function and skin aging. We demonstrate that combined UV + UPM exposure triggers oxidative stress, mitochondrial and DNA damage, senescence, apoptosis, and modulation of melanogenesis in human neonatal melanocytes. In addition, skin explants subjected to the same treatments showed hallmark features of aging, including epidermal thinning, barrier disruption, fibrosis, and altered pigmentation. These findings highlight that melanocytes respond to environmental stress through multiple interconnected mechanisms, potentially affecting both cell survival and pigmentary function. Our model offers a useful platform to study how environmental stressors affect melanocyte function and skin biology, potentially supporting the development of future strategies targeting pigmentation disorders and environmentally driven skin aging.

**Supplementary Information:**

The online version contains supplementary material available at 10.1038/s41598-025-28590-6.

## Introduction

Extrinsic skin aging is primarily driven by chronic exposure to environmental stressors such as ultraviolet (UV) radiation and air pollution^1^. UV radiation, particularly UVA (320–400 nm) and UVB (280–320 nm), penetrates the skin at different depths, eliciting distinct yet overlapping biological responses in both the epidermal and dermal layers^1,2^. UVB, being more energetic due to its shorter wavelength, causes direct DNA damage, membrane disruption, oxidative stress, and triggers apoptosis^3^. Repeated exposure to UV radiation leads to cellular dysfunction, altered melanogenesis, and cumulative tissue damage that accelerates skin aging^4^.

In parallel, urban air pollution—especially exposure to urban particulate matter (UPM)—has emerged as a significant extrinsic factor contributing to the aging process. UPM is a heterogeneous mixture of solid and liquid particles suspended in air, including metals, minerals, organic toxins, tobacco smoke, and allergens^5,6^. These pollutants penetrate the skin barrier and trigger a cascade of oxidative and inflammatory responses, resulting in protein and DNA damage, lipid peroxidation, barrier dysfunction, pigmentation changes, and loss of skin hydration^7,8^.

Melanocytes play a central role in the photoprotective response by producing melanin. However, with aging, melanocytes undergo functional decline and can enter a state of cellular senescence^9^. Cellular senescence serves as a protective tumor-suppressive mechanism, halting the proliferation of damaged or stressed cells and thereby preventing tumor growth. Despite this, their long-term accumulation in tissues can have detrimental effects, contributing to aging and the development of age-related diseases^10^. Senescent cells exhibit characteristic features, including increased expression of cell cycle inhibitors such as p16 and p21, senescence-associated β-galactosidase (SA-β-Gal) activity, altered chromatin organization, and the secretion of pro-inflammatory mediators, collectively known as the senescence-associated secretory phenotype (SASP)^11^. In the skin, senescent melanocytes accumulate with age and influence the tissue microenvironment via SASP, impairing the proliferation and function of neighboring cells, such as keratinocytes, ultimately disrupting tissue homeostasis^12–14^.

In prior investigations, we established a model of melanocyte and fibroblast senescence induced by UVB radiation, identifying compromised proteasomal function and heightened autophagy as pivotal characteristics of UVB-induced senescence^3,15^. In a subsequent study, we demonstrated that the combination of ultraviolet radiation with UPM exacerbates oxidative stress and DNA damage in dermal fibroblasts, thereby shifting the cellular response from senescence to apoptosis. This finding underscores the synergistic impact of environmental co-stressors on the aging of skin^16^.

While the impacts of environmental stressors on skin biology are well-documented, the specific molecular pathways activated in melanocytes by air pollution, particularly under combined exposures, remain insufficiently characterized. In this study, we build upon our previous fibroblast model and apply stressors to melanocytes, combining UVA + UVB and UPM exposure to investigate stress-induced senescence, cellular dysfunction, and dysregulation of melanogenesis. Furthermore, we use ex vivo human skin explants to capture the tissue-level consequences of environmental exposure. This multilayered approach enables a comprehensive investigation into how synergistic environmental insults drive melanocyte dysfunction and contribute to broader mechanisms of skin aging.

## Materials and methods

### Chemicals

Unless stated otherwise, all chemicals were purchased from Sigma-Aldrich (Steinheim, Germany).

### Cell culture and treatments

Primary human neonatal epidermal melanocytes (HNEM) (CellSystems Biotechnologie Vertrieb) were cultured in DermaLife basal medium supplemented with DermaLife M Life Factors (CellSystems Biotechnologie Vertrieb). The cells were derived from neonatal foreskin of Caucasian origin (light pigmentation phototype. All experiments were conducted using the same melanocyte line to ensure consistency across experiments. UV irradiation of cells was performed once daily for four consecutive days, using 7 J/cm^2^ of UV-A and 0.05 J/cm^2^ of UV-B per treatment. The BIO-SUN irradiation system (Vilber) with UV lamps (4 × 30-Watt 365 nm and 2 × 30-Watt 312 nm) was used for irradiation. After the fourth day of treatment (D4), the cells were either used for experiments or allowed to recover and then used for further experiments on day 9 (D9). UPM was acquired from Sigma-Aldrich (NIST1648A) and is a well-characterized standard reference material containing a heterogeneous mixture of polycyclic aromatic hydrocarbons, metals, and other organic compounds. The mean particle size is less than 10 µm, with a distribution reflecting airborne particulate matter. For UPM-only conditions, cells were seeded, and UPM was added to the culture medium immediately after seeding (D0)at a concentration of 5 µg/mL. For combined UV + UPM treatment, UPM (5 µg/mL) was also added on the day of seeding. From the second day onward, cells were irradiated with UVA and UVB, and fresh medium containing UPM was added immediately after irradiation. This treatment cycle was repeated daily until the last day of treatment, after which cells were maintained in fresh medium without UPM. The cell numbers were determined using a CASY Cell Counter (Schärfe System). Cumulative population doublings (cPDLs) were calculated as previously described^17^. Cell morphology and size changes were monitored using a Nikon Eclipse TE300 inverted microscope (Nikon) equipped with light microscopy.

### Cytochemistry for SA-β-galactosidase

To monitor senescence, cells were stained for SA-β-galactosidase activity as described^15,18^. Blue staining, indicative of SA-β-Gal activity, was documented by brightfield microscopy (Nikon Eclipse TE300 microscope connected to a Nikon Digital Sight DS-U2 camera; Nikon). The percentage of positive cells was calculated by dividing the number of positive blue cells by the total number of cells in the field.

### Determination of apoptotic and necrotic cell death by flow cytometry using annexin V/PI staining

Cells were stained with Annexin V/PI to detect apoptotic and necrotic cells according to the manufacturer’s protocol (FITC Annexin V Apoptosis Detection Kit I, BD Pharmingen™). Instead of PI, DAPI staining (1:5000) was used in some cases. The cells were measured using a BD FACS Canto II flow cytometer (BD Biosciences) or LSR Fortressa (Fortessa, Becton Dickinson). The percentage of apoptotic cells was presented as the sum of early-stage (Annexin V-positive/PI-negative or DAPI-negative) and late-stage (Annexin V-positive/PI-positive or DAPI-positive) apoptotic cells. Melanocytes treated with UV + UPM were pre-incubated for 1 h with 30 µM Z-VAD, 40 µM Nec-1 (MedChemExpress), or the combination of both from D1 to D4. As a positive control, HSEM treated with 100 µM cisplatin for 24 h was used, which was also treated with 30 µM Z-VAD to inhibit apoptosis sufficiently as a control. FlowJo software Version 10.9.0 (BD Biosciences) was used to analyze the obtained data.

### Determination of mitochondrial superoxide levels by MitoSOX staining

To assess mitochondrial reactive oxygen species (ROS) levels, the cells were trypsinized and stained with 1 µM MitoSOX green (Invitrogen) for 30 min at 37 °C. The cells were washed with PBS, and fluorescence was measured with a LSR Fortressa (Fortessa, Becton Dickinson). FlowJo software Version 10.9.0 (BD Biosciences) was used to analyze the obtained data.

### Determination of Mitochondrial Membrane Potential by JC-1 Staining

Mitochondrial membrane potential was measured using the fluorescent probe JC-1, as described^19^. For assessment, the populations were divided into two groups based on the percentage of cells with high or low mitochondrial membrane potential, as previously published^19^.

### Measurement of oxygen consumption rates using Seahorse

Oxygen consumption rates were analyzed using the Seahorse XF HS Mini (Agilent Technologies). 10,000 cells were seeded one day before the assay into Seahorse HS Miniplates. The OCR was measured using the Seahorse Cell Mito Stress protocol to assess mitochondrial function, including injections with 1 µM oligomycin, 8 µM FCCP, and 0.5 µM antimycin A/Rotenone.

### Relative mitochondrial DNA (mtDNA)/nuclear DNA (nDNA) ratio

Genomic DNA was isolated from three independent replicates per group using the PureLink™ Genomic DNA Mini Kit (Invitrogen). DNA concentration was assessed using a Nanodrop 2000 (Thermo Scientific) system. 100 ng DNA were used in each qPCR reaction containing either a set of primers binding mtDNA or nDNA as described^19^. Primers for mtDNA (MT-ND4; forward, ACT CTC ACT GCC CAA GAA CT; reverse, GTG TGA GGC GTA TTA TAC CA) and nDNA (GAPDH; primers described below). qPCR was performed using the QuantStudio^TM^7 Flex Real-Time PCR System (ThermoFisher Scientific).

### ATP content

ATP content was assessed using the CellTiter-Glo® Luminescent Assay (Promega). Cells were washed, trypsinized, and resuspended in PBS before the addition of reagents. An equal volume of CellTiter-Glo reagent was added to each sample, and then the samples were mixed on an orbital shaker for 2 min to ensure cell lysis. Plates were then incubated at room temperature for 10 min to stabilize the luminescent signal. Lysate was transferred to a 96-well plate for luminescence measurement using a Victor X5 plate reader (PerkinElmer).

### Detection of specific proteins by immunofluorescence

Cells were seeded in 6-well plates containing coverslips that had been previously coated with poly-D-lysine (Gibco). On the day of collection, the cells were fixed in 4% paraformaldehyde (PFA), permeabilized (0.3% Triton-X, 0.1% sodium citrate in PBS), and incubated with appropriate primary antibodies overnight at 4 °C. After washing, the cells were incubated with secondary antibodies, counterstained with DAPI, and mounted in a fluorescence mounting medium (Dako Cytomation). The following primary antibodies were used: monoclonal mouse anti-γH2AX antibody (#2577S; Cell Signaling Technology), monoclonal mouse anti-TRP1 (#NBP2-61,642; Novus Biologicals), monoclonal mouse anti-MITF (#ab3201; Abcam), rabbit anti-active caspase-3 (#559,565, BD Biosciences), polyclonal rabbit anti-p21 (#2947S, Cell signaling Technology), monoclonal rabbit anti-LaminB1 (ab16048, Abcam), polyclonal rabbit anti-HMGB1 (ab18256, Abcam), monoclonal mouse anti-Ki-67, (sc-23900, Santa Cruz), monoclonal rabbit anti-TOM20 (#42406S, cell singaling Technology), and Alexa Fluor 350 Phalloidin (A22281, Thermo Fisher Scientific). The appropriate Alexa Fluor-conjugated secondary antibodies (Life Technologies) were used. Images were obtained using a confocal scanning system (Cell Voyager CV1000; Visitron Systems). Corrected total cell fluorescence (CTCF) was quantified using the ImageJ software. At least 30 cells were counted for each group in three independent experiments. Mitochondrial fragmentation was analysed by ImageJ in TOM20 images. In addition, senescence-associated heterochromatin foci (SAHF) were quantified by analyzing DAPI-stained images. Cells displaying distinct, condensed nuclear foci characteristic of SAHF were counted and expressed as a percentage of total nuclei.

### Fontana-Masson (FM) staining

FM staining was performed as previously described^15^. Slides were analyzed using Leica DMLS (Leica Mikroskopie & Systeme GmbH). Number of melanosomes per cell was counted using ImageJ software. At least 30 cells were counted in three independent experiments.

### Melanin content

Melanin content measurement was performed as previously described^15^. Absorbance was measured at 492 nm. Melanin content was determined using a standard curve derived from synthetic melanin. All measurements were performed in at least three independent triplicate experiments.

### RNA isolation, cDNA synthesis, and RT-qPCR

RNA was isolated using the RNeasy Mini Kit from Qiagen, according to the manufacturer’s protocol. RNA concentration was measured using a Nanodrop 2000 system (Thermo Scientific). cDNA synthesis was performed using a High-Capacity cDNA Reverse Transcription Kit (Applied Biosystems, Thermo Fisher Scientific) according to the manufacturer’s protocol. Following primer pairs were used for RT-qPCR: Glyceraldehyde 3-phosphate dehydrogenase (GAPDH, housekeeper) (forward, GAG TCA ACG GAT TTG GTC GT; reverse, GAT CTC GCT CCT GGA AGA TG), PGAM5 (forward, GCT ACA TCG TGT GCA GAG CA; reverse, TCT TGT CGG GAG GCA TGA AC), MITF (forward, GGG AGC TCA CAG CGT GTA TT; reverse, ATG GTT CCC TTG TTC CAG CG), and TRP1 (forward, TGG CCA AGT CGG GAG TTT AG; reverse, GTG AGG AGA GGC TGG TTA GC). Quantitative real-time PCR was performed using the QuantStudio^TM^7 Flex Real-Time PCR System with SYBR Green as the dye.

### Preparation of skin biopsies

The study was conducted in accordance with the Declaration of Helsinki. Skin biopsies and their use are covered by ethical permission ECS 1170/2019 issued by the local Ethics committee of the Medical University Innsbruck. Skin tissue was obtained from plastic surgery with the patients’ consent, adhering to the current guidelines of the ethics committee. Biopsies were collected, avoiding sites with visible interferences, such as stretch marks, redness, hair follicle accumulation, and hypo- or hyperpigmented spots. Subcutaneous fat and blood vessels were removed, and the biopsies were cultivated in RPMI 1640 W/GLUTAMAX-I (Gibco, Thermo Fisher) overnight. To mimic physiological skin conditions, the biopsies were positioned at an air–liquid interface, with only the dermal side in contact with the medium. The following day, biopsies were subjected to UV and UPM.

### Histology

Collected tissue was fixed in 4% PFA for 24 h and processed for routine histology to obtain 5 μm serial paraffin sections. General morphology was analyzed by Hematoxylin and Eosin (H&E) staining as previously described^19^. FM staining was used to evaluate pigmentation^15^. Skin biopsies were further stained for Masson´s Trichrome and Periodic Acid Schiff’s (PAS) staining. Slides were analyzed using Leica DMLS (Leica Mikroskopie & Systeme GmbH). Collagen fiber density in Masson´s trichrome and PAS staining was measured using a deconvolution tool in ImageJ, as described in^19^. Additionally, PAS staining was used to measure basal membrane thickness using ImageJ. Paraffin sections obtained from skin explants were subjected to immunofluorescence as previously described^3^, to evaluate epidermal differentiation by using the following antibody: polyclonal rabbit anti-Filaggrin (ab81468; Abcam). Slides were analyzed using a confocal scanning system (Cell Voyager CV1000; Visitron Systems). Epidermal thickness was estimated using ImageJ software in 50 spots across one biopsy. Additionally, the number of pycnotic nuclei in the epidermis was analyzed in relation to the total number of epidermal cells.

### Quantification of FM images of skin biopsies

We applied a standardized image analysis workflow to quantify pigmentation using the Colour Deconvolution 2 plugin in FIJI/ImageJ. First, images were white balanced using Adobe Photoshop to ensure consistent background levels across samples. Following this, color deconvolution was performed using the Colour Deconvolution 2 plugin. This process generated two separate grayscale images representing the individual stain components. We selected the black channel image to quantify melanin content. A defined region of interest (ROI) was created using a square selection. The ROI was then positioned over relevant tissue areas for measurement.

### Lucifer Yellow Diffusion Assay

Skin biopsies were rinsed in PBS, and a 1 mg/mL solution of Lucifer Yellow CH, lithium salt (L0259, Sigma), was topically applied for 2 h. After incubation, the skin was rinsed with PBS, fixed, and processed for embedding. When visualized by fluorescence microscopy, the fluorescent dye labels the outermost layer of the stratum corneum. LY is not particularly prominent in the stratum corneum layer, but it spreads throughout the epidermis and loses its signal. Therefore, we analyzed the percentage of fluorescent-stained area in the stratum corneum using ImageJ.

### Transmission electron microscopy of skin biopsies

Small tissue pieces (2 mm^3^), including epidermis and dermis, were excised from skin biopsies and immediately submersed in 2.5% glutaraldehyde and 2% paraformaldehyde in cacodylate buffer (0.15 M pH 7.4) and further processed as described in^20^. Briefly, samples were post-fixed in 2% osmium tetroxide added with 0.2% ruthenium red in 0.15 M cacodylate buffer, followed by thiocarbohydrazide solution (1% w/v in dH_2_O) and an additional incubation with 2% osmium tetroxide in dH_2_O (double osmium impregnation). Subsequently, samples were incubated in UAR-EMS Uranyl Acetate Replacement Stain (1:4 v/v), followed by Walton’s lead aspartate stain (20 mM lead nitrate in 30 mM L-aspartic acid solution). After progressive dehydration through an ethanol series with a final step in pure acetone, samples were progressively infiltrated in LV Resin (Agar Low Viscosity Resin Kit, Agar Scientific), embedded, and polymerized at 60 °C for 48 h. Resulting blocks were trimmed and sections showing silver interferences were collected on copper grids and imaged under a FEI Tecnai G2 transmission electron microscope operating at 200 kV.

### Statistics

The results are displayed as the mean values of at least three independent experiments ± standard deviation (SD). Differences were compared using one-way ANOVA followed by Tukey’s multiple comparison test. In all graphs, *p < 0.05, **p < 0.01, ***p < 0.001, ****p < 0.0001, and n.s., not statistically significant.

## Results

### UV and air pollution induce cellular senescence in human neonatal epidermal melanocytes

Building on previous work investigating the effects of environmental stressors such as ultraviolet (UV) radiation and air pollution on human dermal fibroblasts^16^, we aimed to explore how these factors affect HNEM and their potential role in skin aging. While it is established that combined stressors accelerate skin aging through mechanisms including apoptosis and cellular senescence, the specific responses of melanocytes under such conditions remain incompletely understood.

**Fig. 1:**
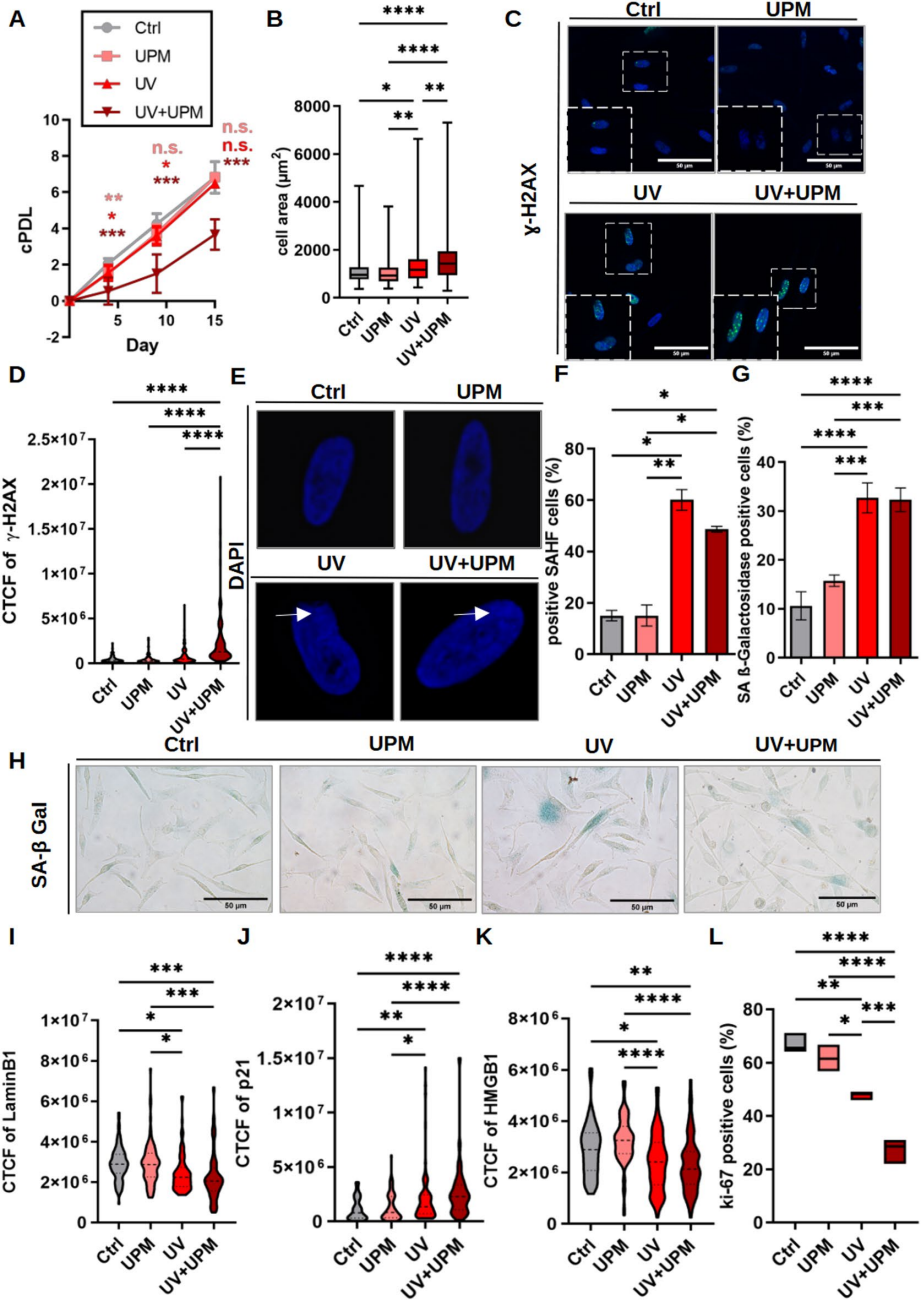
UV and UV + UPM exposure induce senescence-associated features in HNEM. (**A**) Proliferation of HNEM assessed by cPDL over time. (**B**) Measurement of cell area on D9. (**C**) Representative γ-H2AX staining of DNA damage foci. Scale bar: 50 µm. (**D**) Quantification of γ-H2AX immunofluorescence corrected total cell fluorescence (CTCF) on D9. (**E**) DAPI staining in melanocytes to detect SAHF. White arrows indicate SAHF. (**F**) Percentage of SAHF-positive nuclei on D9. (**G**) Quantification of SA-β-Galactosidase staining on D9 of the experiment. (**H**) Representative pictures of SA-β-Gal staining. Scale bar: 50 µm. (**I**) Lamin B1 CTCF on D9. (**J**) p21 (**K**) HMGB1 CTCF on D9. (**L**) Ki-67 staining on D9 to check proliferation. Data are presented as means ± SD from at least three independent biological replicates. Statistical analysis was performed using one-way ANOVA. *p < 0.05, **p < 0.01, ***p < 0.001, ****p < 0.0001.

To address this, we exposed HNEM to UVA + UVB (hereafter referred to as UV), urban particulate matter (UPM), or their combination (UV + UPM) for four consecutive days, following the protocol described in^16^. Proliferation analysis revealed that neither UPM nor UV treatment alone significantly affected melanocyte proliferation compared to the controls. However, cells exposed to the combined UV + UPM treatment exhibited significantly reduced proliferative capacity (Fig. 1A).

By D9, five days after the last treatment, melanocytes exposed to UV or UV + UPM displayed characteristic senescence-associated morphological changes, including cellular enlargement and flattening (Fig. 1B), along with markers of DNA damage, such as γ-H2AX foci and SAHF (Fig. 1C–F). Approximately 30% of cells exposed to UV or UV + UPM were SA-β-Gal-positive, compared to UPM-treated and control cells (Fig. 1G–H). Additionally, analysis of established senescence markers revealed increased expression of the cyclin-dependent kinase inhibitor p21 alongside decreased levels of Lamin B1, HMGB1, and the proliferation marker Ki-67 (Fig. 1I–L, Supp. Fig. 1, and Suppl. Fig. 2A).

Together, these results suggest that while UPM alone does not appear to induce senescence in melanocytes under our experimental conditions, UV exposure is associated with a senescent response that may be enhanced by co-treatment with UPM. These findings suggest a potential role of environmental factors in promoting melanocyte senescence and are consistent with previous observations in human dermal fibroblasts.

### Concomitant treatment of UV and UPM impairs mitochondrial integrity in melanocytes

Mitochondrial dysfunction—encompassing alterations in dynamics, morphology, and function- represents a hallmark of cellular senescence. Dysfunctional mitochondria produce excessive ROS, triggering apoptosis or senescence^21^. Given this critical relationship between mitochondrial health and cellular fate, we investigated how UPM, UV, and UV + UPM exposure affect mitochondrial function in melanocytes.

**Fig. 2:**
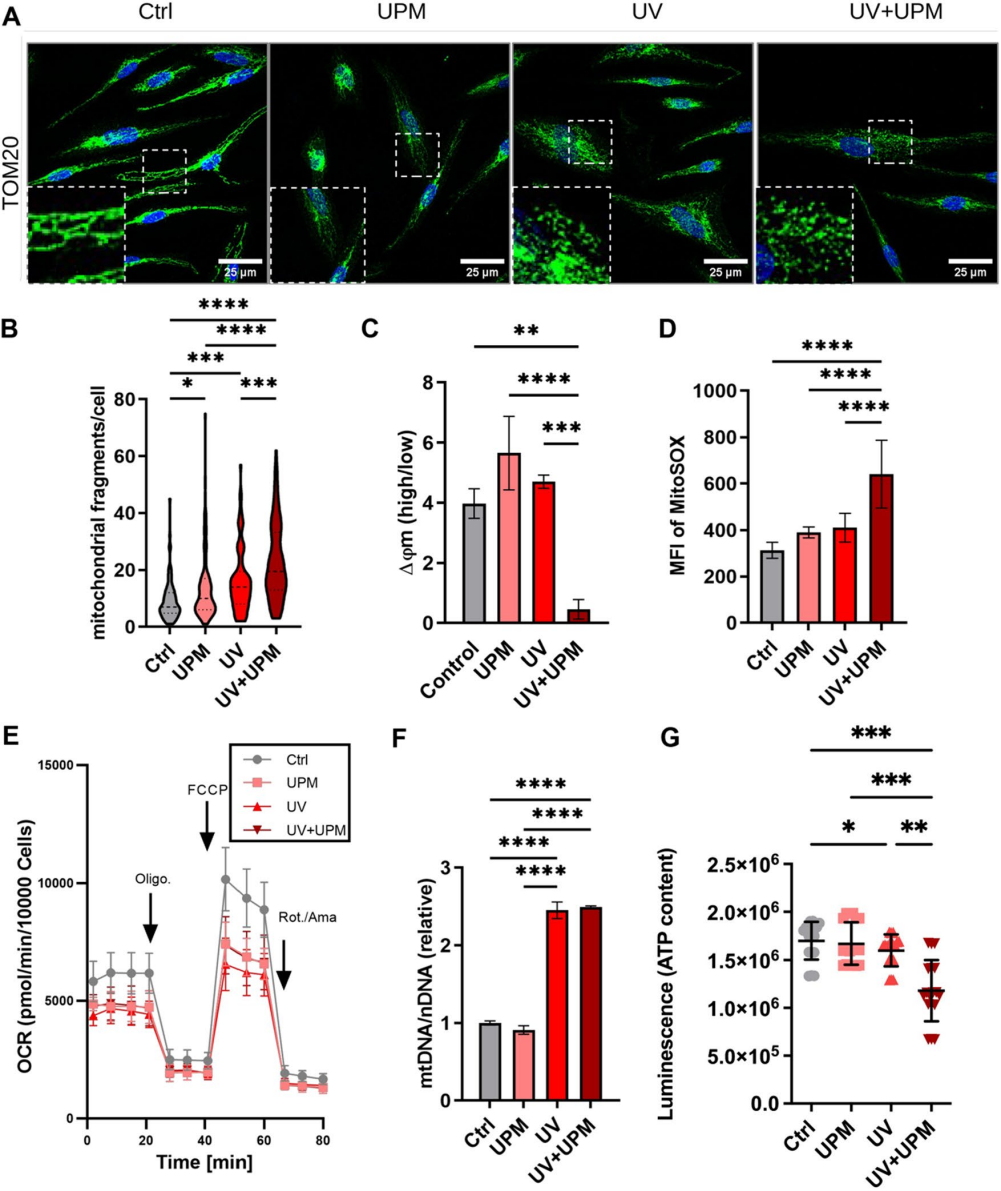
UV and UV + UPM exposure impair mitochondrial morphology and function in melanocytes. (**A**) Representative images of TOM20 immunofluorescence staining at D9. Insets highlight mitochondrial fragmentation. (**B**) Quantification of mitochondrial fragments per cell. (**C**) Mitochondrial membrane potential (ΔΨm), assessed by JC1 staining. (**D**) Mitochondrial ROS production, measured by MitoSOX mean fluorescence intensity. (**E**) Seahorse XF Analyzer assessment of oxygen consumption rate (OCR) following sequential injections of oligomycin, FCCP, and rotenone/antimycin A. (**F**) qPCR quantification of mitochondrial DNA (mtDNA) copy number normalized to nuclear DNA. (**G**) ATP quantification by luminescence. Data are shown as mean ± SD from three independent biological replicates. FACS data were collected from three independent biological and technical replicates. Statistical analysis was performed using one-way ANOVA. *p < 0.05, **p < 0.01, ***p < 0.001, ****p < 0.0001.

Mitochondrial network analysis revealed increased fragmentation across all treatment groups, particularly in UV− and UV + UPM-treated cells (Fig. 2A,B, Supp. Figure 2B). To assess mitochondrial function, we measured mitochondrial membrane potential, which was significantly reduced in UV + UPM-treated melanocytes (Fig. 2C). This was accompanied by elevated mitochondrial ROS levels in the same group (Fig. 2D). Mitochondrial oxygen consumption analysis showed reduced basal and maximal respiratory capacity following all treatments, with the most substantial effects seen in the UV and UV + UPM treatments (Fig. 2E, Supp. Fig. 2C). In parallel, we observed an increase in the relative mtDNA/nDNA ratio, suggesting mitochondrial accumulation upon cellular senescence (Fig. 2F). Finally, ATP levels were significantly decreased in UV- and UV + UPM-treated melanocytes (Fig. 2G), indicating impaired mitochondrial function. These findings suggest that environmental stressors may compromise mitochondrial integrity and function in melanocytes.

### UV + UPM induces apoptosis and non-classical cell death pathways

Although both UV and UV + UPM treatments resulted in comparable senescence phenotypes, only UV + UPM-exposed melanocytes showed a pronounced decrease in cPDL. This disparity suggested that additional cell fate mechanisms, beyond senescence, contribute to the reduced cell numbers during the stress phase. To elucidate the mechanisms underlying the reduced cell numbers observed specifically under UV + UPM conditions, we conducted a comprehensive investigation of cell death pathways.

**Fig. 3:**
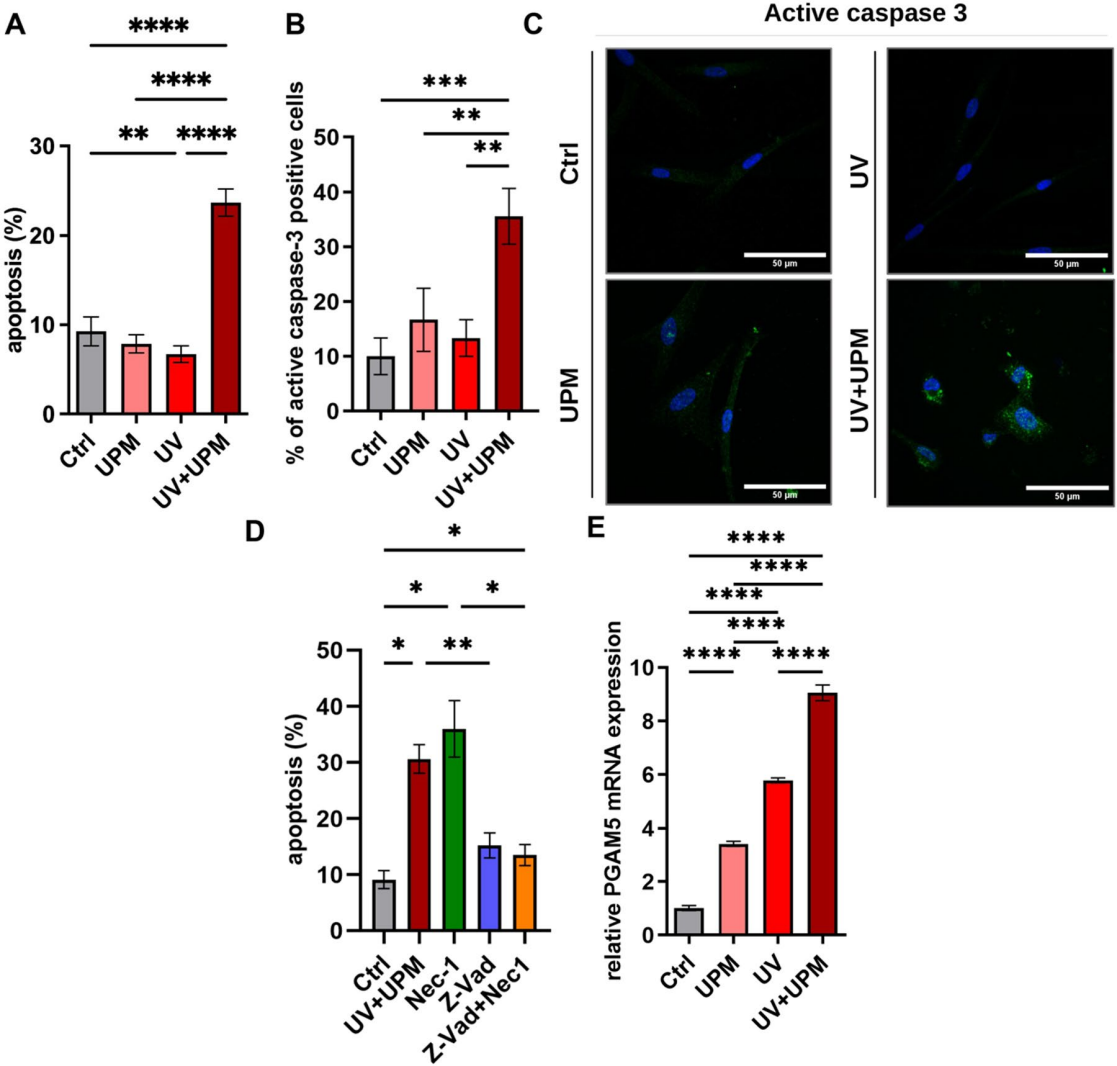
UV + UPM induces apoptosis and non-classical cell death pathways. (**A**) Quantification of apoptosis on D4 by Annexin V staining. (**B**) Quantification of the percentage of positive cells for active caspase-3 on D4. (**C**) Representative images show active caspase-3 staining (green) and DAPI (blue) on D4. Scale bar: 50 µm. (**D**) Quantification of apoptosis by Annexin V staining following pharmacological inhibition of apoptosis (Z-Vad), necroptosis (Nec-1), and combined inhibition upon UV + UPM treatment. (**E**) Relative mRNA expression of PGAM5 on D4. Data are presented as mean ± SD from three independent biological and technical replicates. Statistical analysis was performed using one-way ANOVA. *p < 0.05, **p < 0.01, ***p < 0.001, ****p < 0.0001.

Annexin V staining performed on D4, the final day of treatment, revealed a significant increase in apoptosis exclusively in the UV + UPM group (Fig. 3A). This finding was corroborated by elevated levels of active caspase-3 in UV + UPM-treated cells on D4 (Fig. 3B,C), indicating activation of the classical apoptotic pathway. Importantly, these apoptotic markers were absent at the later time point (D9) (Suppl. Fig. 3A–C), suggesting that apoptosis occurs primarily during the stress phase rather than when senescence is established. These findings suggest that the reduced cell numbers in UV + UPM-treated melanocytes result from a combination of increased apoptosis and senescence induction.

To explore whether alternative cell death pathways contribute to the fate of melanocytes following UV + UPM exposure, we employed pharmacological inhibitors targeting distinct cell death mechanisms. Treatment with Z-VAD (a pan-caspase/apoptosis inhibitor) or Nec-1 (a necrosis inhibitor) failed to fully rescue cell viability in UV + UPM-exposed melanocytes (Fig. 3D). This incomplete rescue, coupled with the absence of significant necrosis as assessed by PI/DAPI staining (data not shown), strongly indicated the involvement of non-classical cell death pathways.

Given the elevated oxidative stress observed in UV + UPM-treated cells and the incomplete protection offered by classical cell death inhibitors, we investigated the potential occurrence of oxeiptosis—a recently characterized ROS-dependent, caspase-independent cell death pathway^22^. Oxeiptosis is mediated by Mitochondrial Serine/Threonine Protein Phosphatase 5 (PGAM5), a mitochondrial phosphatase implicated in mitochondrial dynamics and oxidative stress responses. PGAM5 expression was upregulated across all treatment groups, with significantly higher in UV + UPM-treated cells (Fig. 3E, Suppl. Fig. 3D).

These data suggest that, beyond classical apoptosis, oxidative stress-induced cell death mechanisms—potentially including oxeiptosis—may contribute to the reduced viability of melanocytes following UV + UPM exposure. This potential multi-modal cell death response could help explain the more pronounced decrease in cell numbers observed under UV + UPM conditions compared to UV treatment alone, despite similar senescence phenotypes.

### Environmental stressors dynamically regulate melanogenesis in human melanocytes

**Fig. 4:**
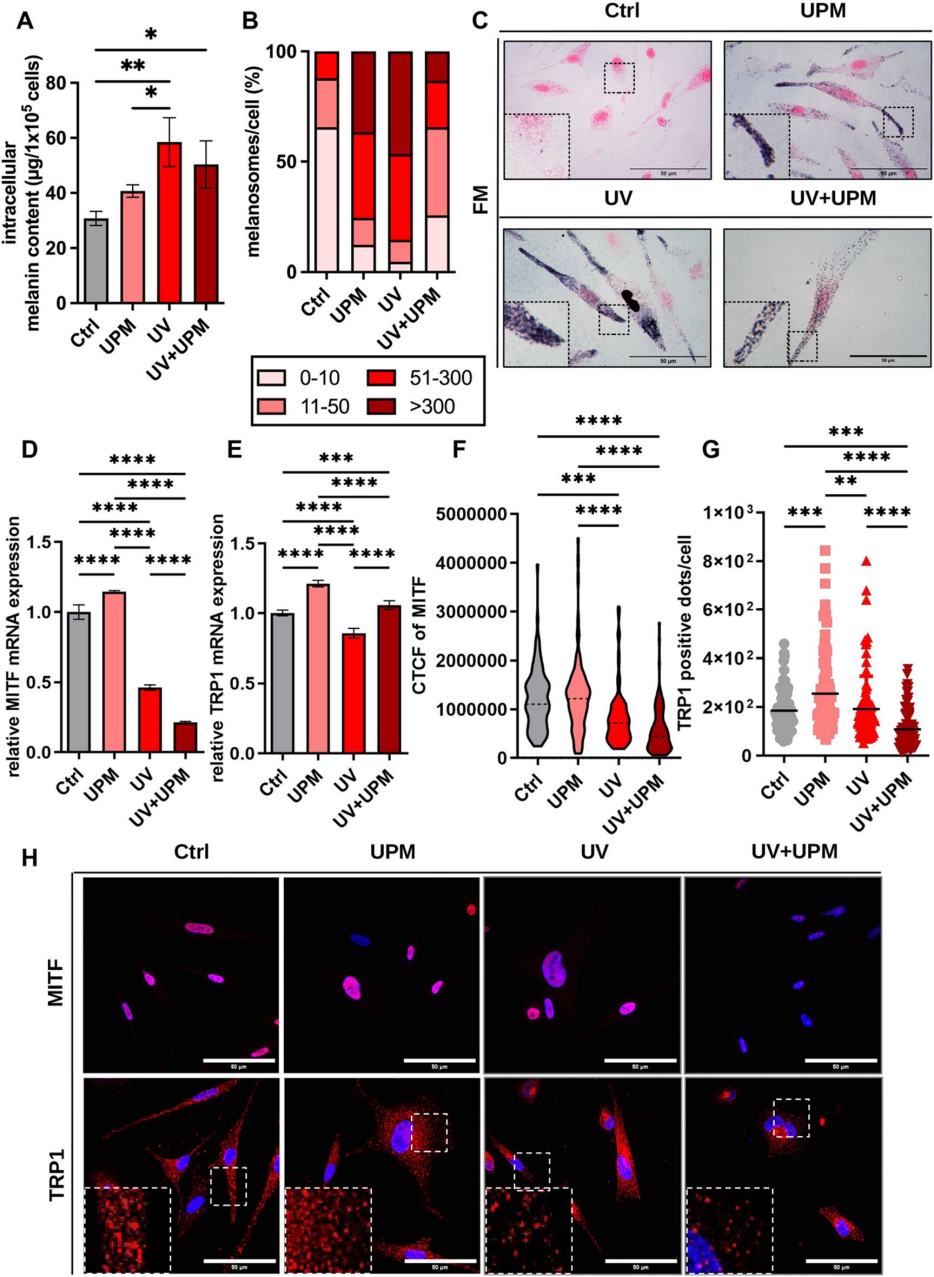
Environmental stressors modulate melanogenesis in human epidermal melanocytes. (**A**) Quantification of intracellular melanin content after treatment with UPM, UV, or UV + UPM on D9. (**B**) Distribution of melanosomes per cell in FM-stained cells on D9 was categorized into four groups (0–10, 11–50, 51–300, > 300). (**C**) Representative FM staining showing melanin accumulation (black deposits) in treated melanocytes on D9. Insets highlight differences in melanosome density and localization. Scale bar: 50 µm. (**D**–**E**) Relative mRNA expression of melanogenesis markers MITF and TRP1 at D4. (**F**–**G**) Quantification of CTCF for MITF and TRP1-positive dots per cell based on immunofluorescence on D4. (**H**) Representative immunofluorescence images of MITF (top) and TRP1 (bottom). Nuclei were counterstained with DAPI (blue). Insets highlight TRP1-positive structures. Scale bars: 50 µm. Data are presented as mean ± SD from three independent biological replicates. Statistical analysis was performed using one-way ANOVA. *p < 0.05, **p < 0.01, ***p < 0.001, ****p < 0.0001.

Given the protective role of melanin in shielding the skin from UV radiation, we examined how environmental stressors modulate melanogenesis in NHEM. Quantification of intracellular melanin revealed that UV and UV + UPM treatments significantly increased melanin content by D9, while UPM alone showed a non-significant upward trend (Fig. 4A). These findings were further supported by FM staining, which showed increased melanosome accumulation in all treated groups (Fig. 4B,C).

To understand the regulatory basis of this response, we assessed the expression of key melanogenesis-related genes at D4 (stress phase). Microphthalmia-associated transcription factor (MITF), the master transcription factor for melanogenesis, was significantly downregulated in UV and UV + UPM-treated cells, whereas it was upregulated by UPM alone (Fig. 4D). Tyrosinase-related protein-1 (TRP1), in contrast, was upregulated by UPM and UV + UPM, but suppressed by UV alone (Fig. 4E). Immunofluorescence analysis confirmed these transcriptional changes at the protein level, as demonstrated by reduced MITF and TRP1 signal intensity in both UV and UV + UPM-treated cells (Fig. 4F–H; Supp. Fig. 4A,B).

Notably, by D9, when cells had established senescence, MITF and TRP1 mRNA expression levels were restored or upregulated across all treatment groups (Supp. Fig. 5A–E and Supp. Fig. 6A,B). This rebound in senescent cells corresponded with the observed increases in intracellular melanin content and melanosome accumulation per cell by D9 (Fig. 4A–C).

These findings suggest that melanogenesis may undergo dynamic regulation in response to environmental stressors in our model system, potentially characterized by an initial suppression during acute stress, followed by compensatory upregulation upon the establishment of senescence.

### Environmental stressors induce structural and cellular changes in human skin explants

**Fig. 5:**
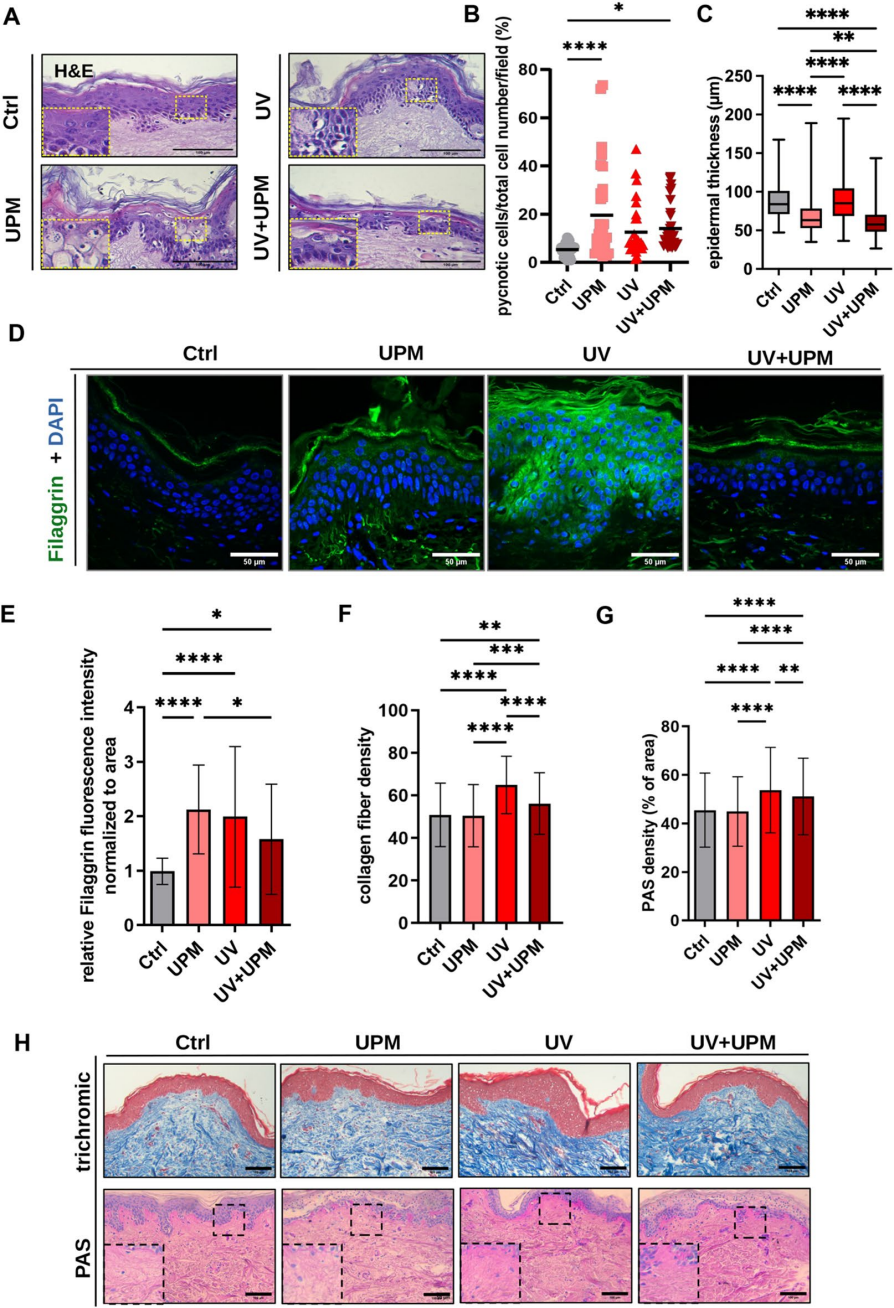
Environmental stressors induce structural and molecular alterations in human skin explants. (**A**) Representative H&E staining of skin sections. Insets highlight pycnotic nuclei. Scale bar: 100 µm. (**B**) Quantification of pycnotic nuclei as percentage of total epidermal cells. (**C**) Measurement of epidermal thickness. (**D**) Representative immunofluorescence images of skin sections stained for Filaggrin (green) and DAPI (blue). Scale bars: 50 µm. (**E**) Quantification of relative fluorescence intensity normalized to area for Filaggrin. (**F**) Quantifying collagen fiber density in the dermis. (**G**) Quantification of PAS-positive staining density in the dermis (% area). (**H**) Representative images of Trichrome (top) and PAS (bottom) staining of dermal compartments. Insets highlight dermal architecture. Scale bar: 100 µm. Data are presented as mean ± SD from at least three independent donors. Statistical analysis was performed using one-way ANOVA. *p < 0.05, **p < 0.01, ***p < 0.001, ****p < 0.0001.

To validate our in vitro findings in a physiologically relevant system, we employed human skin explants, which retain the native tissue architecture and cellular complexity of the skin. The experimental treatments mirrored those used for cultured melanocytes, except for a higher UPM dose (50 µg/day) to account for limited diffusion in tissue. Skin explants were harvested and processed for histological analysis after the last day of treatment (D4). H&E staining revealed an increased number of pycnotic nuclei in the epidermis across all treatment groups, indicating keratinocyte apoptosis (Fig. 5A,B). Notably, epidermal thickness, an established feature of skin aging, was significantly reduced in UPM- and UV + UPM-treated explants (Fig. 5C). Interestingly, analysis of filaggrin expression showed increased fluorescence intensity in all treated groups (Fig. 5D,E, Supp. Fig. 7A) with ectopic expression in suprabasal layers upon UV exposure, suggesting aberrant or premature expression of this late differentiation marker.

To assess structural remodeling in the dermis, we performed Masson’s Trichrome and PAS staining. Both UV and UV + UPM exposure increased collagen fiber density in the papillary dermis (Fig. 5F–H), indicative of fibrotic remodeling. These alterations likely reflect maladaptive repair responses to chronic environmental stress. These results suggest that UV and UPM exposure may compromise epidermal integrity and induce dermal remodeling in our ex vivo model, potentially reflecting features associated with skin aging.

### UV + UPM impairs skin integrity and promotes post-inflammatory pigmentation features

**Fig. 6:**
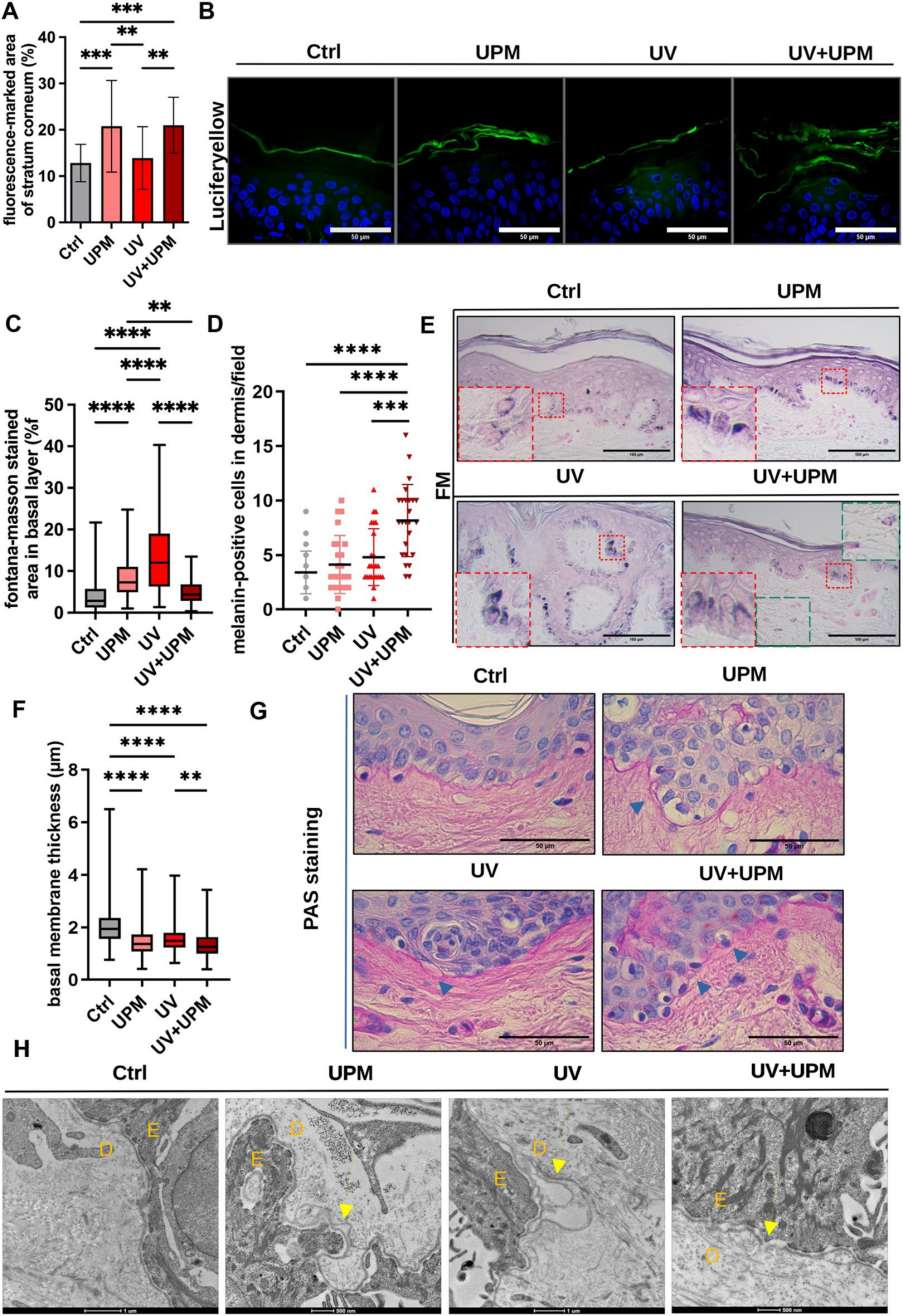
Environmental stressors compromise skin barrier function and basal membrane integrity and modulate pigmentation patterns. (**A**) Quantification of LY penetration across the stratum corneum. (**B**) Representative images of LY staining (green). Nuclei counterstained with DAPI (blue). Scale bars: 50 µm. (**C**) Quantification of epidermal melanin by FM staining. (**D**) Quantification of melanin-positive cells in the dermis per field. (**E**) Representative FM staining shows basal epidermal pigmentation (red insets) and ectopic dermal melanin in UV + UPM-treated skin (green inset). Scale bars: 100 µm. (**F**) Measurement of basal membrane thickness using PAS staining. (**G**) Representative images of PAS-stained explants. Black arrows indicate thinning or disruption of the basal membrane. Scale bars: 50 µm. (**H**) TEM images showing ultrastructural alterations in the dermal–epidermal junction. D = dermis; E = epidermis. Data are presented as mean ± SD from at least three independent donors. Statistical analysis was performed using one-way ANOVA. *p < 0.05, **p < 0.01, ***p < 0.001, ****p < 0.0001.

To assess the broader tissue-level consequences of environmental stress exposure, we evaluated epidermal barrier permeability. Increased Lucifer yellow fluorescence revealed increased stratum corneum permeability following UPM and UV + UPM exposure, indicating compromised barrier integrity (Fig. 6A,B).

FM staining showed increased epidermal pigmentation in UV- and UPM-treated explants. In contrast, UV + UPM-treated skin displayed ectopic melanin accumulation in the dermis (Fig. 6C–E), a hallmark of post-inflammatory hyperpigmentation (PIH) associated with leakage of melanin to the dermal compartment due to basement membrane disruption. To explore this further, we analyzed the thickness of the basal membrane via PAS staining. Basement membrane thickness was reduced in all treatment groups, with UV + UPM showing the most pronounced decrease (Fig. 6F,G). Transmission electron microscopy confirmed structural breaks at the dermal–epidermal junction only in the UV + UPM group (Fig. 6H).

Collectively, these results suggest that environmental stressors weaken the skin barrier and basement membrane integrity in our ex vivo model, potentially causing pigment to leak into the dermis and possibly contributing to pigmentation disorders resembling PIH.

## Discussion

This study provides new insights into the combined effects of UV radiation and urban particulate matter (UPM) on melanocyte biology and skin aging. Our findings suggest that UPM synergistically enhances UV-induced cellular dysfunction, leading to senescence, mitochondrial impairment, alternative cell death pathways, and dysregulated pigmentation. These cellular changes translate to tissue-level alterations in human skin explants, providing a useful experimental model for studying environmentally induced skin aging.

While UV radiation has been well-characterized as a driver of melanocyte senescence, the role of air pollution in melanocyte aging has remained largely unexplored. Our results suggest that UPM acts as a co-stressor, potentially amplifying UV-induced damage without being inherently senescence-inducing on its own. This finding may have implications for understanding skin aging in urban environments, where populations are simultaneously exposed to both stressors. The synergistic effect likely stems from complementary damage pathways: UV causes direct DNA damage while UPM impairs cellular repair mechanisms, shifting the balance from adaptive responses toward cell death.

Mitochondria play a central role in regulating cellular senescence by controlling energy metabolism, redox balance, and apoptotic signaling^23^. Mitochondrial dysfunction emerged as a critical feature of the melanocyte response to UV and UPM exposure. Acute oxidative stress induces mitochondrial fission and dysfunction^24^. Senescent melanocytes exhibit metabolic reprogramming, characterized by increased glycolysis, a shift that is associated with mitochondrial dysfunction^25^. In UV + UPM-treated melanocytes, we observed disrupted mitochondrial membrane potential, increased ROS, and reduced ATP levels, suggesting impaired oxidative phosphorylation (OXPHOS)^26^, a phenomenon also observed in keratinocytes and fibroblasts^27–29^. This metabolic dysfunction may influence not only energy homeostasis but also the decision between senescence and alternative cell death pathways^30^.

Mitochondrial damage is a well-known upstream trigger of apoptosis, and indeed, caspase activation confirmed the involvement of classical apoptosis in the UV + UPM condition. Our data also suggest that apoptosis alone may not fully explain the observed cell loss. PI/DAPI staining ruled out significant necrosis, indicating the possible involvement of alternative, non-apoptotic cell death pathways. Given the pronounced oxidative stress induced by UV + UPM, we considered oxeiptosis—a recently characterized, ROS-driven, caspase-independent cell death mechanism—as a plausible contributor^22,30^. Although preliminary transcriptional evidence (e.g., PGAM5 upregulation) supports this, further functional studies, including verification of other key oxeiptosis-associated proteins, are needed to determine its precise role in our model.

The consequences of mitochondrial stress extend beyond energy metabolism and cell fate decisions—they also intersect with melanocyte-specific functions such as pigmentation. Melanogenesis enables melanocytes to protect skin from UV damage. This process is tightly regulated and complex, involving various activation and feedback mechanisms^31^. MITF serves as the primary transcription factor responsible for producing essential melanogenesis-related proteins and enzymes, and functions as a “UV-protection timer” in melanocytes, coordinating stress responses and pigmentation through dynamic, oscillatory expression. MITF is expressed rhythmically with a 24-h periodicity in the presence of circadian clock protein BMAL1^32^. UVB exposure every 48 h enhances pigmentation and reduces DNA damage compared to daily exposure, highlighting a frequency-dependent protective mechanism^33^. Our model revealed diminishing expression of MITF and TRP1 during the stress phase, suggesting a temporary suppression of pigment synthesis, which could serve as an adaptive response to acute stress. This was followed by increased melanin accumulation at later stages, likely reflecting melanosome buildup rather than active neomelanogenesis. Beyond pigmentation, MITF is essential for maintaining genome integrity: its depletion increases DNA damage by downregulating DNA repair processes^34^ and induces senescence via direct activation of p16^INK4A^ and p21^Waf1/cip1^, leading to retinoblastoma protein hypophosphorylation and cell cycle arrest—a crucial step in proper melanocyte differentiation and cellular senescence^35,36^. Thus, MITF appears to act as a central cellular regulator, and its suppression under stress likely contributes to melanocyte senescence and dysfunction.

In parallel, mitochondrial dynamics regulate pigmentation. Mitofusin-2, known for mediating mitochondrial fusion and inter-organelle contacts, forms physical tethers between mitochondria and melanosomes, which are essential for melanosome maturation and function^37,38^. Furthermore, the mitochondria-melanosome interaction is hypothesized to play an important role in vitiligo, a depigmentation disorder^39,40^. This suggests a crucial role for mitochondrial function, mitophagy, and melanosome maturation, indicating that oxidative stress-induced mitochondrial dysfunction, particularly under UV + UPM conditions, may delay or impair melanosome development. However, the mitochondria-melanosome axis during cellular senescence induction remains unclear.

Skin explant data provided additional support for our in vitro findings at the tissue level. Concomitant UV + UPM exposure induced hallmark features of aged skin, including epidermal thinning, increased pyknotic nuclei, impaired barrier function, and altered pigmentation. While UV or UPM alone increased pigmentation, their combination did not lead to further epidermal pigment accumulation; instead, we observed melanin leakage into the dermis, resembling the pathology of PIH and solar lentigos^41,42^. We confirmed a disruption of the basement membrane that impaired pigment distribution under stress conditions. In nevi, clusters of dermal senescent melanocytes can stimulate epithelial hair follicle stem cells, causing them to exit quiescence and enhancing hair regeneration^43^. Additionally, loss of sprouty homolog 1 protein in keratinocytes triggers a tanning-like response by driving melanocyte stem cell migration into the epidermis through the p53/SCF/c-KIT axis^44^. This raises the question of whether the melanosome-like structures we observed in the dermis represent only pigment leakage or reflect melanocyte migration into the dermal compartment, a matter that remains to be clarified.

Importantly, the effects of UV + UPM on skin explants extend beyond melanocytes. In particular, senescent fibroblasts have been identified as key modulators of extracellular matrix (ECM) remodeling. In the context of skin aging, it has been well described that extrinsic factors such as tobacco smoke and UV exposure lead to reduced collagen production and elevated matrix degradation, primarily through the upregulation of matrix metalloproteinases^19,45^. However, recent studies have highlighted a dynamic interplay between fibroblast senescence and ECM remodeling in age-related fibrosis. The concept of ‘fibroageing’ describes how ECM stiffening, driven by glycation, crosslinking, and disrupted matrix turnover, leads to a mechanical microenvironment that supports the persistent activation of pro-fibrotic pathways^46^. Senescent fibroblasts secrete matrix-modifying enzymes and pro-inflammatory SASP factors, reinforcing a feedback loop that perpetuates both fibrosis and senescence^47,48^. In our model, UV and UPM exposure contribute to ECM remodeling that resembles a fibrosis-like phenotype. This is supported by previous findings showing that UV + UPM exposure induces senescence in dermal fibroblasts along with mitochondrial dysfunction, impaired autophagy, and elevated apoptosis^16^. These findings support the growing perspective that senescence-ECM remodeling could provide innovative therapeutic strategies for addressing environmentally induced skin aging.

Furthermore, melanocytes are notably responsive to signals from neighboring cells, including senescent fibroblasts, which release SASP factors that can influence melanocyte behavior and pigmentation^49–51^. In skin spots such as senile lentigo, senescent fibroblasts accumulate and contribute to hyperpigmentation due to the absence of stromal cell-derived factor-1 in their SASP profile. Notably, targeted elimination of these fibroblasts reduced pigmentation, emphasizing the SASP as a promising therapeutic target for pigmentary disorders^51^. This highlights the significance of dermal–epidermal communication in our UV + UPM model, suggesting that its role in environmentally induced skin aging necessitates further investigation.

Although using melanocyte monocultures and skin explants together provides complementary insights, this study has some limitations that should be considered when interpreting the results. First, we did not evaluate melanocyte-specific markers in skin tissue, which limits our ability to directly link the skin-aging phenotype to melanocyte dysfunction. Additionally, melanocyte–fibroblast interactions have not been studied, which are crucial for a comprehensive understanding of paracrine communication and its role in stress-induced pigmentation and senescence. Furthermore, although UPM was applied topically using a physiologically relevant air–liquid interface, the extent to which UPM particles penetrate the intact epidermal barrier was not evaluated in this study. We also did not assess the antioxidant defense capacity of melanocytes or skin tissue under treatment conditions. As impaired antioxidant responses can lead to excessive ROS accumulation, future work should investigate how redox buffering systems are altered by UV and UPM exposure. Moreover, although our data suggest that multiple cell death mechanisms may contribute to reduced melanocyte viability, we did not assess other non-classical pathways, such as ferroptosis, which are increasingly linked to oxidative stress and environmental insults. While we identified potential involvement of oxeiptosis through PGAM5 expression analysis, functional validation of this pathway was not performed. Future work should investigate and validate the involvement of these alternative stress-induced death mechanisms. From an experimental design perspective, while our exposure protocol of repeated daily treatments over four consecutive days was designed to mimic chronic exposure to mild doses of environmental stressors, the timeframe may still be limited compared to the decades of environmental exposure that contribute to human skin aging. The UPM concentrations used (5 μg/mL for cell culture and 50 μg/day for skin explants) were selected based on previous literature but may not represent physiologically relevant doses for all urban environments, as particulate matter composition and concentration vary significantly between locations and seasons. Moreover, we employed discrete UVA and UVB wavelengths instead of a solar simulator to enable controlled and reproducible exposure conditions, consistent with our previous fibroblast studies. While this approach allowed us to analyze wavelength-specific biological effects, we acknowledge that solar simulators provide a closer representation of natural sunlight and should be used in future studies to validate our findings under more physiologically relevant exposure spectra. Another limitation is that only melanocytes from individuals with light skin phototypes (Caucasian neonatal origin) were used. Pigmentation status influences oxidative stress responses and senescence trajectories; therefore, future studies should include melanocytes from individuals with darker skin phototypes and those from aged donors. Lastly, our experiments were conducted on Caucasian neonatal melanocytes. The response of darker skin phototypes or aged melanocytes to UV and UPM exposure may differ significantly, and future studies should investigate pigmentation-dependent susceptibility and senescence trajectories under environmental stress. Additionally, the ex vivo skin explant model, while maintaining tissue architecture better than isolated cell cultures, lacks normal vascular perfusion, immune cell interactions, and systemic factors that influence skin responses in vivo.

In conclusion, our findings provide new insights into the interaction between UV radiation and urban pollution, which disrupts melanocyte function and compromises skin structure. We captured cellular and tissue-level responses, suggesting that multilayered mechanisms may contribute to extrinsic skin aging. The establishment of these complementary models not only enhances our understanding of individual stressor effects but also creates a foundation for investigating intercellular crosstalk—particularly between fibroblasts and melanocytes—under environmental stress conditions. This experimental model may serve as a valuable tool for dissecting the contribution of specific cell types and could support the development of targeted strategies for pigmentation disorders and senescence-associated skin deterioration.

## Supplementary Information

Supplementary Material 1

## Data Availability

The data that support the findings of this study are available from the corresponding author upon reasonable request.
