# Supplementary material for: Synergistic interplay between UV and urban particulate matter exposure induces melanocyte senescence and contributes to human skin aging
Source: Sci Rep. 2025 Dec 29;15:44893. doi: 10.1038/s41598-025-28590-6 (PMC12749940; doi:10.1038/s41598-025-28590-6)
Supplement: Supplementary file 1 — Supplementary Material 1 [file 41598_2025_28590_MOESM1_ESM.docx]

**Supplementary Figures:**


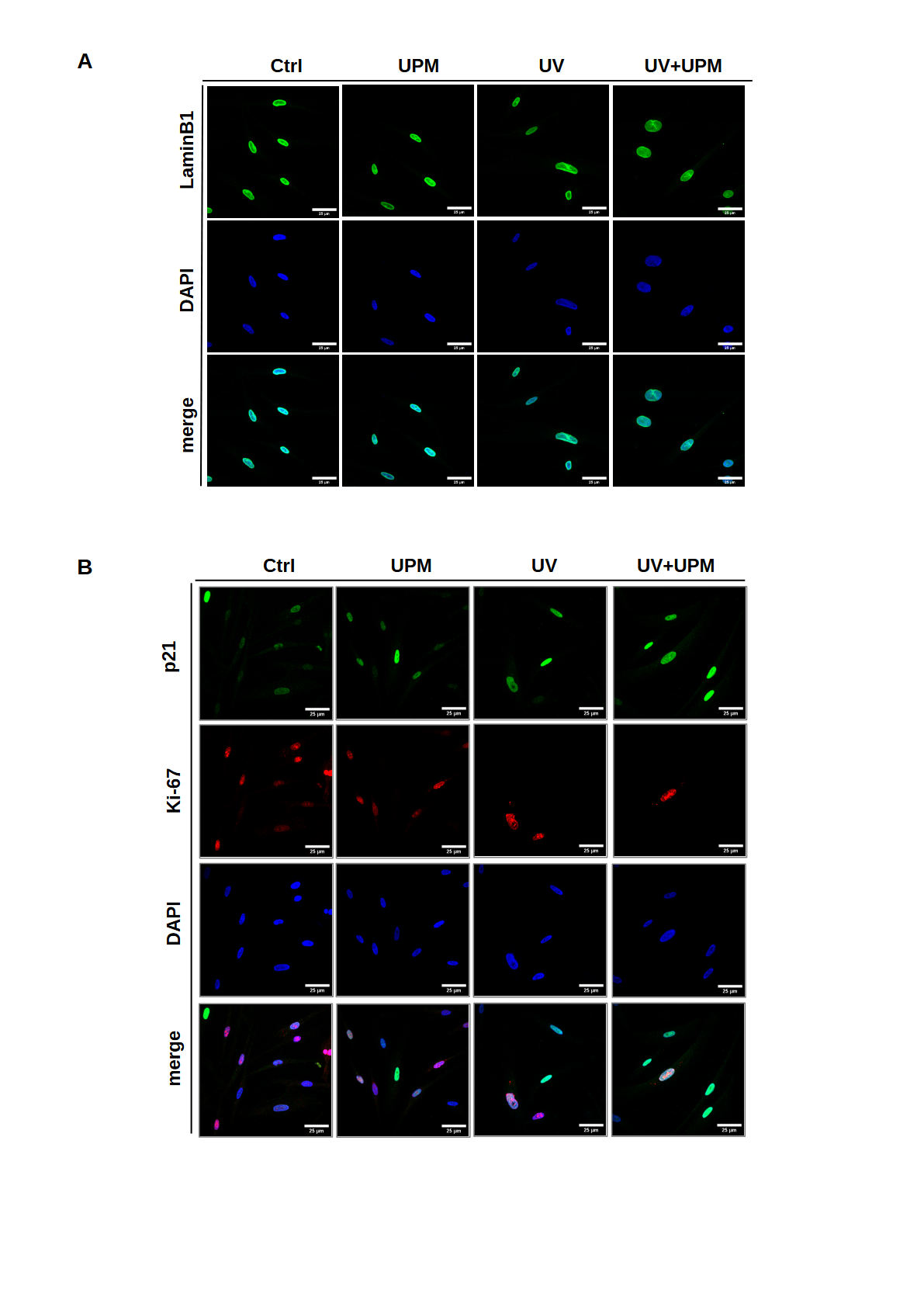


**Supplementary Figure 1.**
**Senescence and proliferation markers in melanocytes following UPM and UV exposure.**
**(A)** Immunofluorescence staining for Lamin B1 (green) and nuclear DAPI (blue). **(B)** Co-staining of p21 (green) and Ki-67 (red) to evaluate cell cycle arrest and proliferation status, respectively. Nuclei were counterstained with DAPI (blue). Scale bars: 25 μm.


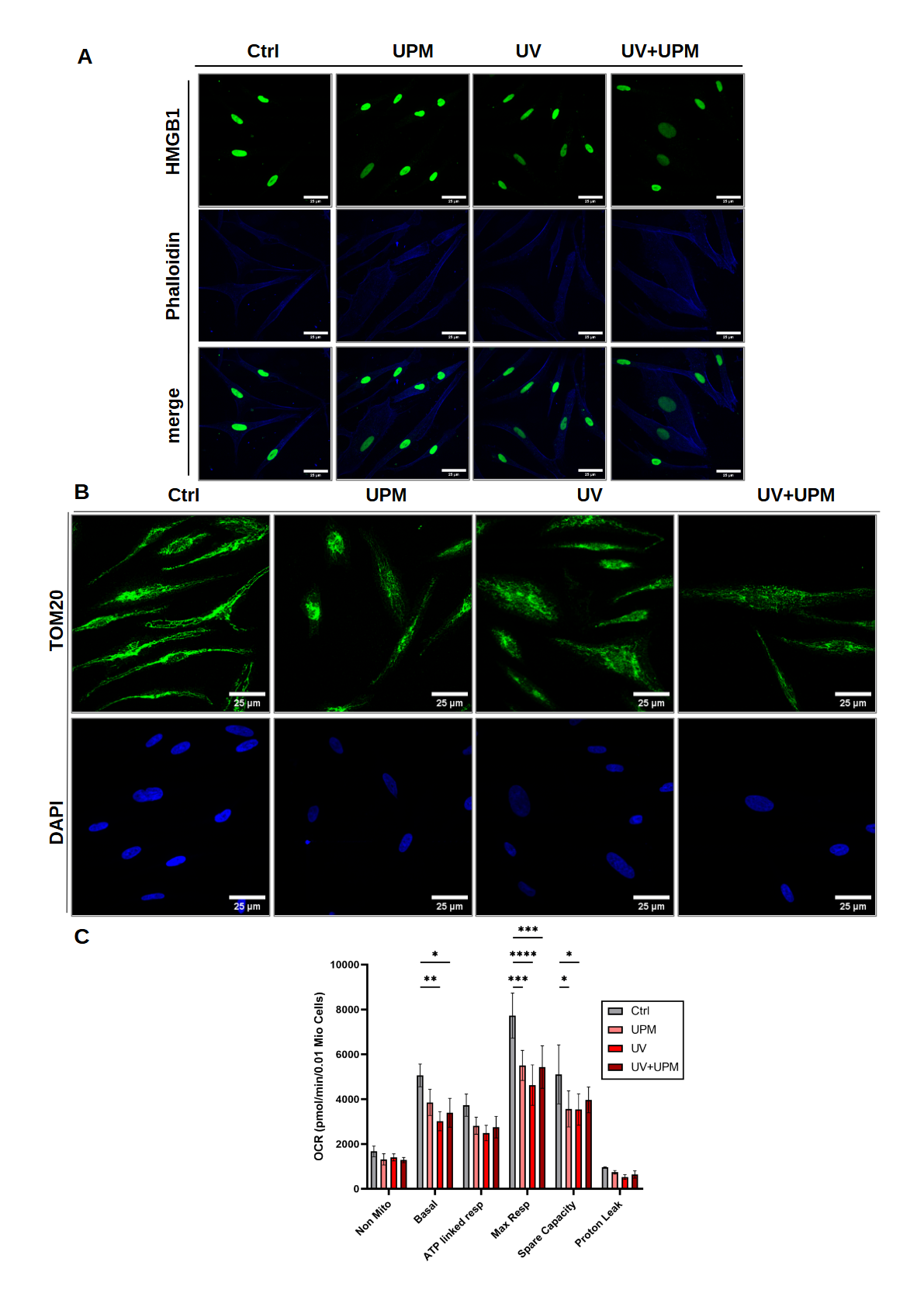


**Supplementary Figure 2.**
**Assessment of stress response and mitochondrial function in melanocytes following UPM and UV exposure.**
**(A)** Immunofluorescence staining of HMGB1 (green) and phalloidin (blue) to visualize nuclear translocation and cytoskeletal organization. Scale bar: 25 µm. **(B)** Mitochondrial morphology was assessed using TOM20 staining (green); nuclei were counterstained with DAPI (blue). Scale bar: 25 µm. **(C)** Seahorse-based quantification of oxygen consumption rate (OCR) across mitochondrial respiratory parameters. Data are presented as mean ± SD; statistical significance indicated (*p < 0.05, **p < 0.01, ***p < 0.001, , ****p < 0.0001).


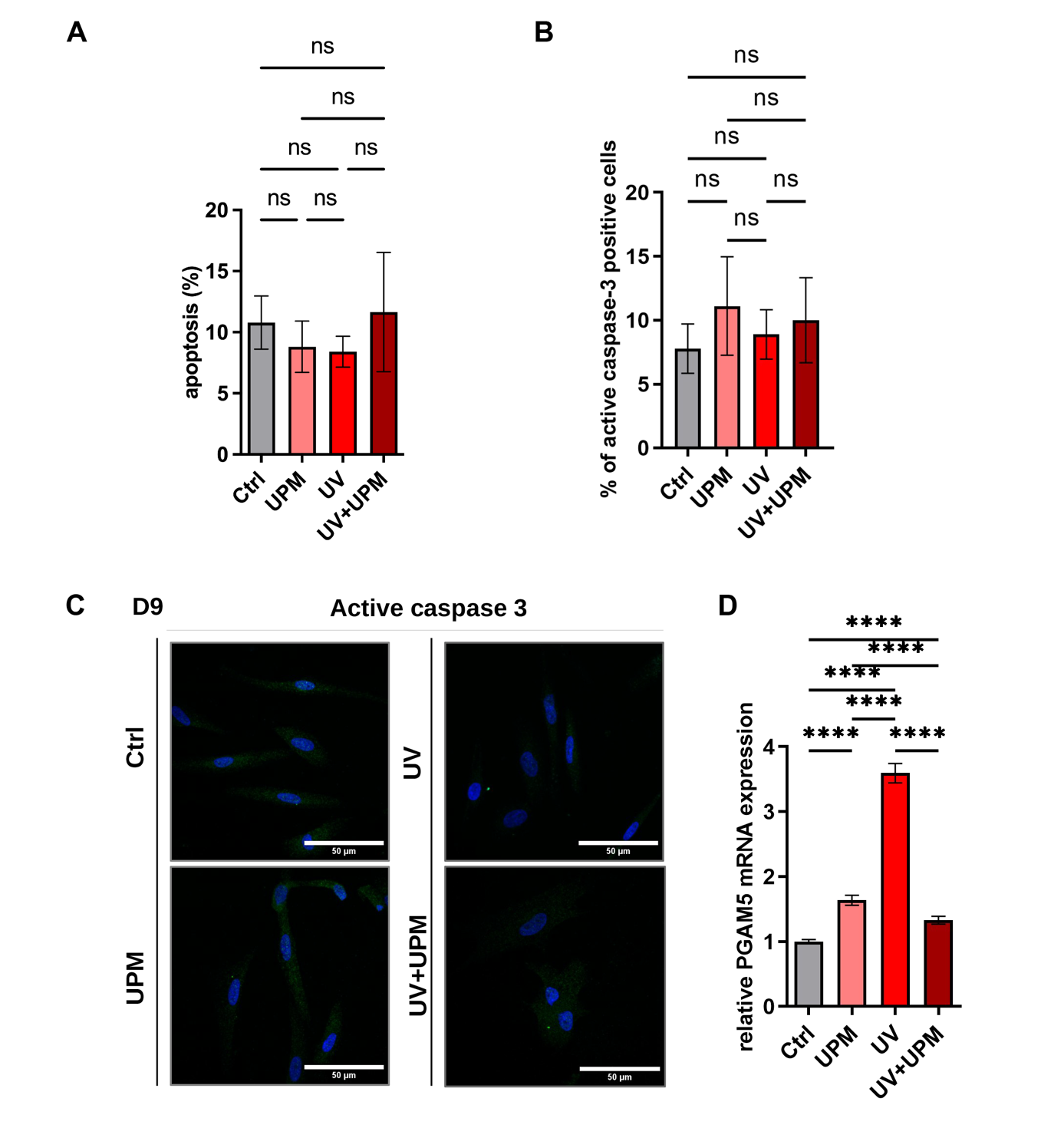


**Supplementary Figure 3.**
**UPM and UV exposure induce apoptosis.**
**(A)** Quantification of apoptotic cells on D9 by Annexin V staining. **(B)** Quantification of the percentage of positive cells for active caspase-3 on D9. **(C)** Representative images show active caspase-3 staining (green) and DAPI (blue) on D9. Scale bar: 50 µm. (D) Relative mRNA expression of PGAM5 on D9. Data are presented as mean ± SD; statistical analysis was performed using one-way ANOVA. *p < 0.05, **p < 0.01, ***p < 0.001, ****p < 0.0001.


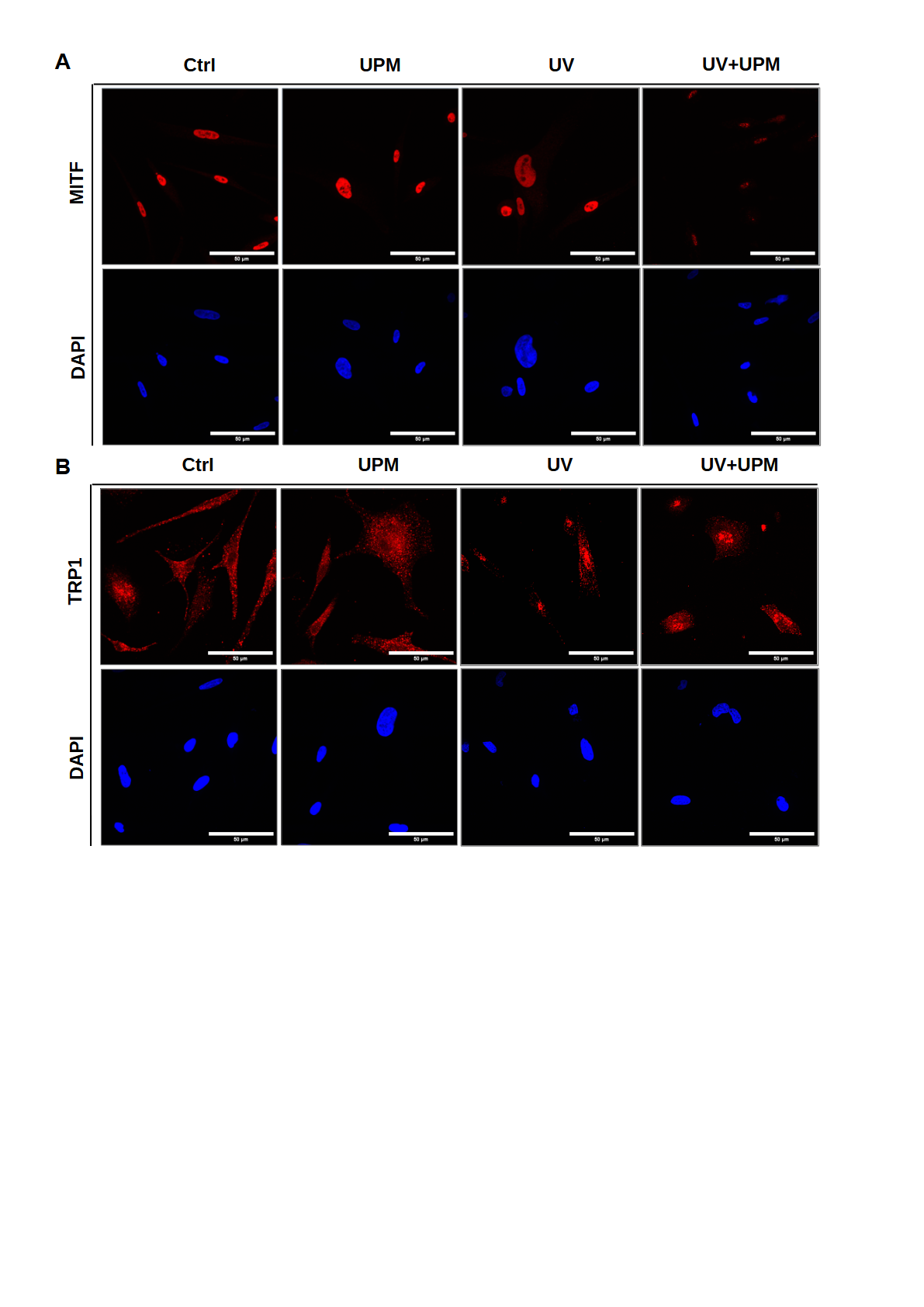


**Supplementary Figure 4.**

**Immunofluorescence analysis of pigmentation-related proteins in melanocytes following environmental stress exposure.**

**(A)** Representative images showing MITF (red) and nuclear DAPI staining (blue) in control (Ctrl), UPM-, UV-, and UV+UPM-treated HNEM. Scale bar: 50 µm. **(B)** Representative images showing TRP1 (red) and DAPI (blue) in the same conditions. Scale bar: 50 μm.

**
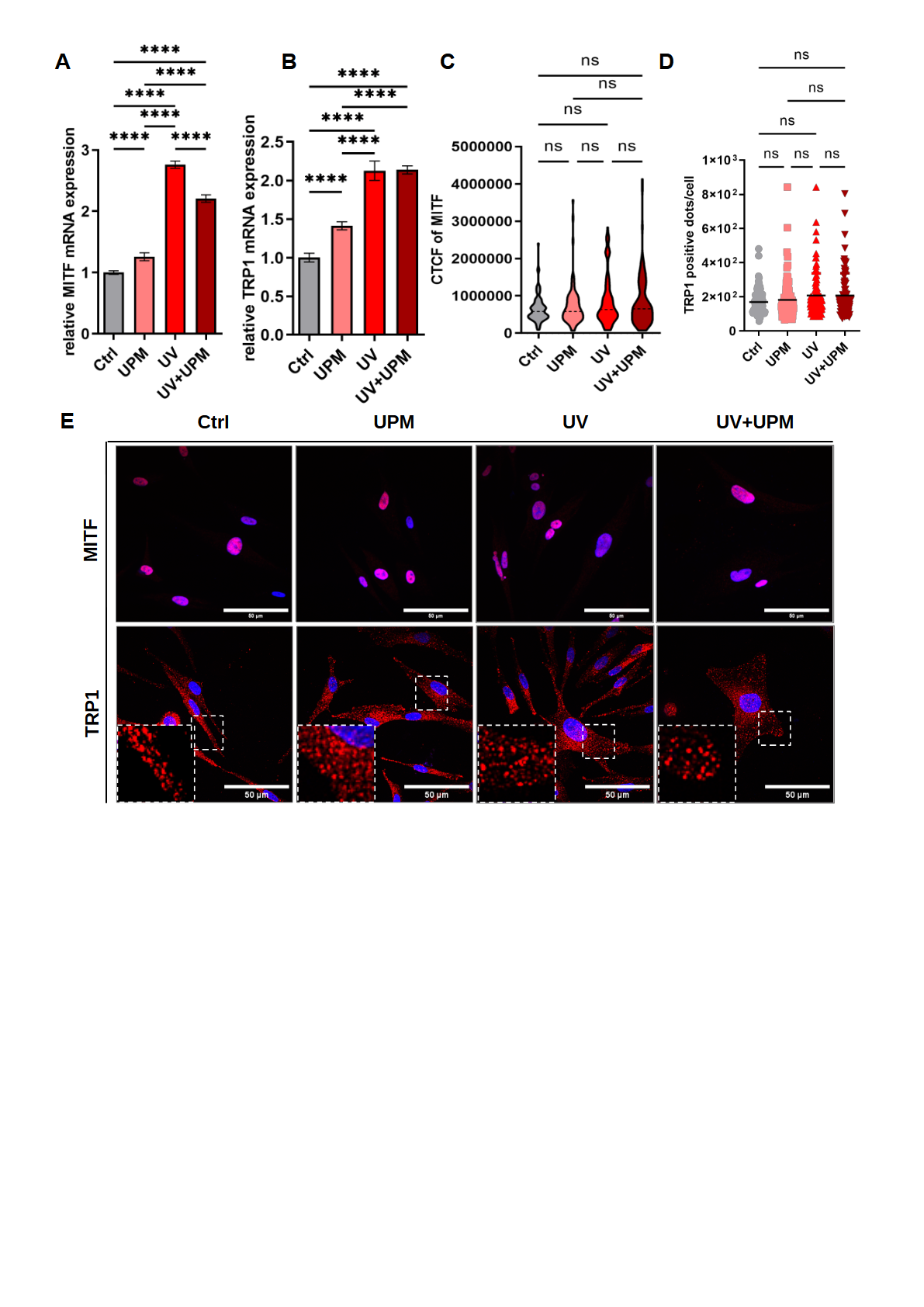
Supplementary Figure 5.**

**Environmental stressors modulate melanogenesis in human epidermal melanocytes.**

**(A)** Relative mRNA expression of melanogenesis markers MITF and **(B)** TRP1 measured at D9. **(C)** Quantifying corrected total cell fluorescence (CTCF) for MITF and **(D)** TRP1-positive dots per cell based on immunofluorescence analysis. **(E)** Representative immunofluorescence images of melanocytes stained for MITF (upper panel) and TRP1 (lower panel). Nuclei are counterstained with DAPI (blue). Insets highlight increased puncta TRP1 structures. Scale bars = 50 µm. Data are presented as mean ± SD; statistical analysis was performed using one-way ANOVA. *p < 0.05, **p < 0.01, ***p < 0.001, ****p < 0.0001.


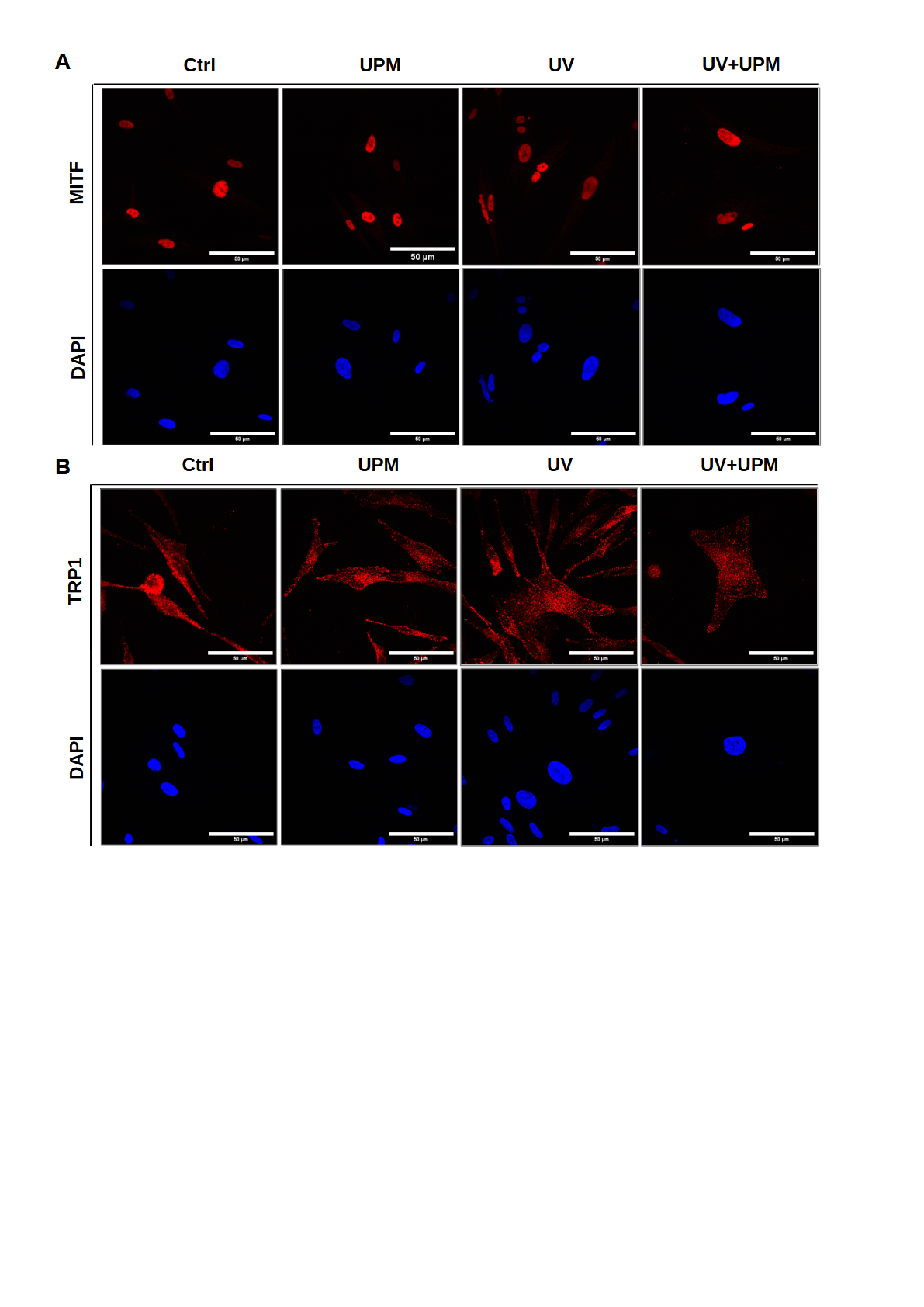


**Supplementary Figure 6.**

**Immunofluorescence analysis of pigmentation-related proteins in melanocytes following environmental stress exposure on D9.**

**(A)** Representative images showing MITF (red) and nuclear DAPI staining (blue) in control (Ctrl), UPM-, UV-, and UV+UPM-treated HNEM. Scale bar: 50 µm. **(B)** Representative images showing TRP1 (red) and DAPI (blue) in the same conditions. Scale bar: 50 μm.


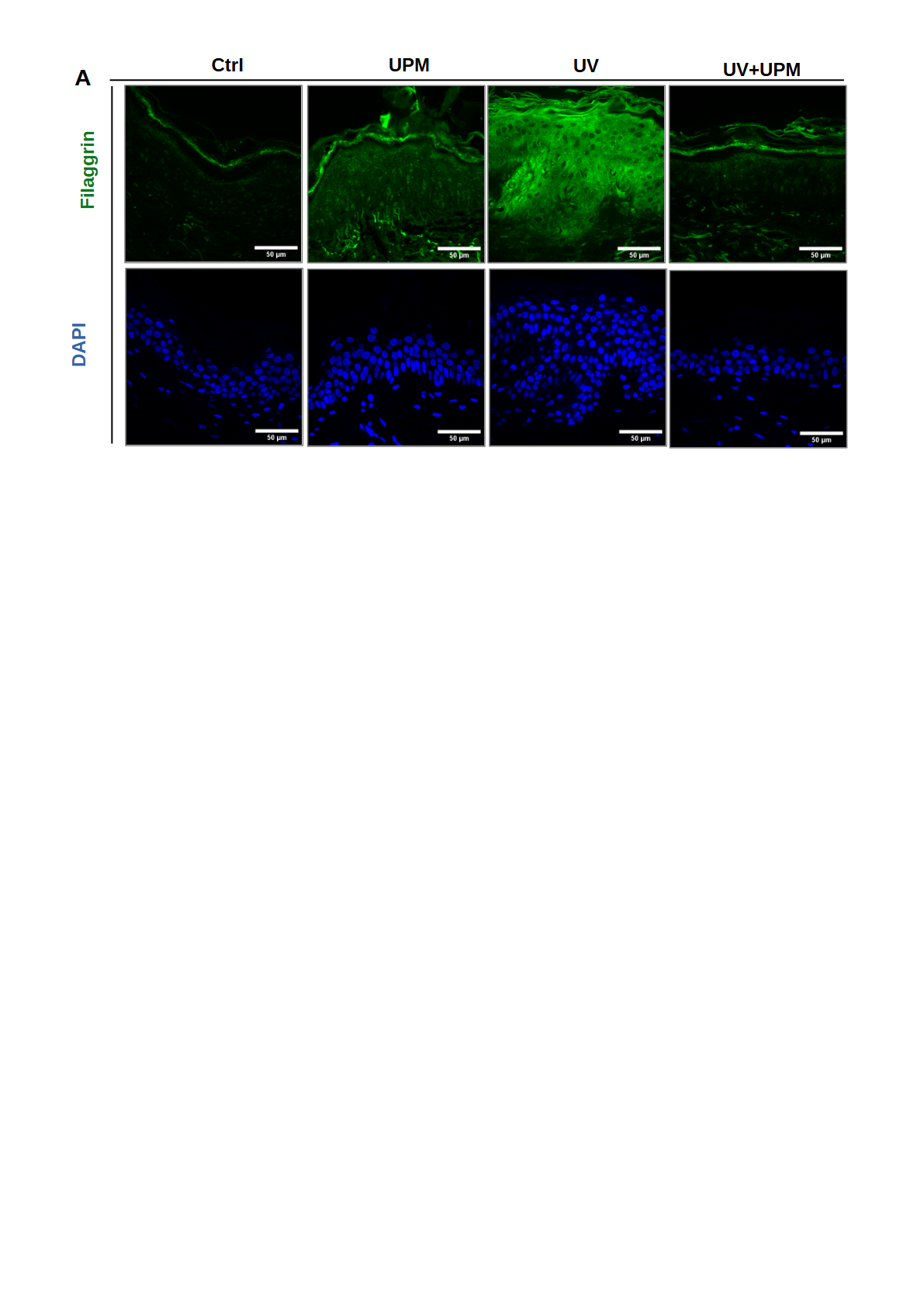


**Supplementary Figure 7.**

**Immunofluorescence staining of epidermal differentiation protein filaggrin in skin explants exposed to environmental stressors.**

**(A)** Representative images showing expression of Filaggrin (green) and nuclear DAPI (blue) in control (Ctrl), UPM-, UV-, and UV+UPM-treated skin explants. Scale bars: 50 μm.
